# *Ruta chalepensis* L. Essential Oil Has a Biological Potential for a Natural Fight against the Pest of Stored Foodstuffs: *Tribolium castaneum* Herbst

**DOI:** 10.1155/2020/5739786

**Published:** 2020-08-27

**Authors:** Mariame Najem, Mohamed Bammou, Lamia Bachiri, El Houssine Bouiamrine, Jamal Ibijbijen, Laila Nassiri

**Affiliations:** Environment & Soil Microbiology Laboratory, Moulay Ismail University of Meknes, Faculty of Sciences, Meknes B. P: 11201, Morocco

## Abstract

Chemical pesticides used against insect pests of stored food have adverse effects on both health and the environment. So, the present study aims to evaluate the insect repulsive and insecticidal power of *Ruta chalepensis* L. essential oil (EO) from the region of Oulmes (Central plateau of Morocco); the ultimate objective is to develop a biological and ecological control strategy against pests. Thus, the EO obtained by hydrodistillation from the aerial parts of *Ruta chalepensis* L. was identified by GC-MS; its repellent and fumigant toxicity effects on adults of *Tribolium castaneum* Herbst were, respectively, investigated by the preferential area method on a filter paper and the inhalation test. The insecticide power was estimated by determining the percentage of mortality as a function of the duration of exposure and concentration of the EO. The essential oil obtained is characterized by the dominance of 2-undecanone (64.35%), piperonyl piperazine (11.9%), 2-decanaone (5.12%), 2-dodecanone (4.52%), decipidone (3.9%,) and 2-tridecanone (2.36%). This EO is endowed with a very repulsive power belonging to class V, which is strongly due to its majority compound 2-undecanone. The dose 0.038 *μ*l/ml gave a repellent power of 100% after 15 min. The tests also revealed a considerable insecticidal effect, which reached 100% after 48 hours at a dose of 0.62 *μ*l/ml. The calculation of the lethal dose causing 50% mortality (LD50) and the lethal times after which there is 50% mortality (LT50) allowed deducing that the insecticidal effect of *Ruta chalepensis* L. is time- and dose-dependent. Hence, the effectiveness of *Ruta chalepensis* L. EO attests that it can constitute a healthy alternative to fight against *Tribolium castaneum* Herbst.

## 1. Introduction

Faced with the increasing demand for food and in order to prevent its insecurity around the world, it is necessary to reduce losses before and after harvesting crops [1]. Indeed, during the storage phase, foodstuffs, in particular, cereal grains and legumes, which constitute the food base in several countries, are attacked by insects; the production loss is great when the storage period is long and could be total if there is no protection [2, 3].

The methods used to limit losses in stocks are generally based on the use of chemical pesticides reputed to be effective; however, the intensive use of these products causes contamination of the biosphere and trophic chains and induces chronic poisoning among consumers [4, 5].

Even more, over the generations, the appearance of resistant insect pests is noted; also, an eradication of many nontarget species such as auxiliary fauna is caused. Therefore, the World Health Organization (WHO) has banned the use of certain chemical insecticides [6] and others are in the process of being prohibited.

Therefore, it became imperative to use alternatives which, while ensuring effective protection of foodstuffs during storage, would not cause health problems or any other harm to consumers or to the environment [7].

In this context, numerous studies are increasingly invested in plants to isolate or identify secondary metabolites which are able to have insecticidal, repellent, or antipalatable activity against insects. Thus, some research has shown that essential oils have several properties allowing them to be considered as biopesticides in alternative strategies with the aim of limiting the use of synthetic pesticides; these biopesticides, as they are biodegradable, are considered as products with low ecological impact [8].

The toxic effect of essential oils is based, in addition to the variability of phytochemical compounds, on other factors such as the point of entry of the toxin. Indeed, the bioactivity of essential oils is directly affected by its chemical composition and its content of active compounds, which can vary considerably, even within the same species. The variability factors of this composition involve the part of the plant used, the phenological state of the plant, the harvest period, the place of harvest, and the conditions of the growing environment [9]. Generally, essential oils can be inhaled, ingested, or absorbed through the skin by insects [9]. Thus, one of the properties of essential oils that deserve particular interest is their fumigant activity against insects, as it provides pest control during storage without the need for direct application of the compound to insects and without direct contact of the compound with the stored products.

In this context, the present study reports for the first time the chemical composition of the essential oil of the aerial part of *Ruta chalepensis* from the region of Oulmes (Central plateau of Morocco) and evaluates its repellent and fumigant activity against adults of *Tribolium castaneum*, a cosmopolitan pest of stored grain products, as part of future control strategies against these pests.

## 2. Materials and Methods

### 2.1. Biological Material

#### 2.1.1. The Plant Used

The samples of the aerial part (stems, leaves, and flowers) of *Ruta chalepensis* L. are collected during May 2018, around the Oulmes region (Central plateau of Morocco).

In fact, the region of the central plateau abounds in thermo-Mediterranean plant formations structured by cork oak, holm oak, oleaster, thuja, and atlas pistachio; at the beginning, we carried out a survey to locate the *Ruta* and it turned out that this was at the level of the barbary thuja formation, developed on a superficial skeletal soil.

Then, we penetrated right into this formation in order to avoid the margins (usually degraded) and the boundaries between two neighboring formations which are often heterogeneous; once in an apparently floristically homogeneous surface “SAFH,” we make transection in the four directions, following ropes of 5 meters each. While moving along each rope, the *Ruta* plants encountered were collected and placed in a bag bearing the number and direction of the transect in question. This operation was repeated at five points, chosen at random from the “SAFH.”

**Figure 1:**
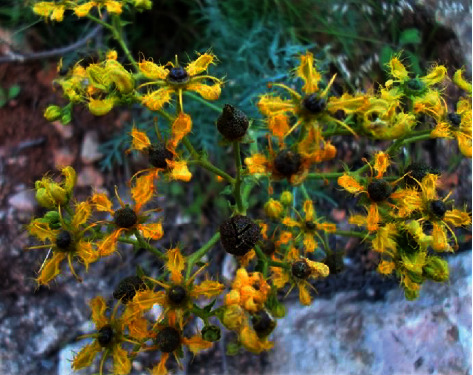
*Ruta chalepensis* L.

The samples, carefully placed in coolers, were taken directly back to the laboratory. Subsequently, its identification was confirmed in the Soil and Environment Microbiology Laboratory at the Faculty of Sciences in Meknes, and a reference specimen (Figure 1), carefully prepared and labeled, was deposited under the number: MICSOL- Rc 01.

Freshly harvested and washed, the plant was left to dry in the shade in a ventilated place; then, the parts to be used were stored in clean bags away from light and moisture.

#### 2.1.2. The Insect Used

We worked with infested soft wheat collected in the cereal storage warehouses of the local market, representing the largest ones in the region; we wanted our study to focus on the *Tribolium castaneum* colonies infecting these premises, with a view to developing a biopesticide that could be subsequently applied there. After sieving and using entomological forceps, we separated the insects recovered on the basis of their morphological resemblance. Also, for taxonomic identification and confirmation, a binocular magnifying glass was used to visualize the main morphoanatomical features.

The identification of *Tribolium castaneum* Herbst was based on several morphological criteria. Our starting point was insects larger than 1 mm and not moths; insects with antennae but without distinct caudal appendages were selected for the next stages of identification. At this stage, insects with a size less than 10 mm, a head not extended by a rostrum, and straight antennae without distinct elbows were selected. Next, insects with a head that is clearly visible in dorsal view and with lateral edges of the pronotum that are not toothed were retained. Then, we selected the insects which have antennae terminated by more or less distinct clubs and which are deprived of tubercles at the level of the anterior angles of the pronotum. To avoid confusion between *Tribolium castaneum* and *Tribolium confusum*, the antennal clubs and the space between the eyes were examined. Retained insects have an antennal club abruptly enlarged and a narrow space between the eyes [10–12].

After identification, we established an initial population of *Tribolium castaneum* for mass rearing. Adult individuals were placed in 1-liter glass jars containing 100 g of the soft wheat which was crushed since *Tribolium castaneum* is a secondary granivorous insect that feeds, both in the adult stage and in the larval stage, mainly on broken wheat [13]. These jars were placed in an incubator set at a temperature of 30°C ± 1°C and relative humidity of 70% ± 5% [14]. In order to avoid the overcrowding phenomenon, adults were regularly transferred to new jars, thus ensuring new infestations. For all tests, adults aged 10–14 days were used [15].

### 2.2. Extraction of *R*. *chalepensis* Essential Oil

The essential oil was extracted from aerial parts of *Ruta chalepensis* L. by hydrodistillation using a Clevenger-type device. Distillation was carried out by boiling, for 3 h, 100 g of fresh plant material with 1000 ml of water in a 1-liter flask surmounted by a 60 cm long graduated column connected to a condenser [16]. The vapor loaded with essential oils, passing through the refrigerant, condenses and falls in the graduated burette, thus allowing the volume collected to be read directly. Water and oil separate by a difference in density; indeed, the condensed vapor produces two phases: an organic phase containing the essential oil and an aqueous phase (aromatic hydrosol) which contains a nonnegligible quantity of oil either in solubilized form or in the form of finely dispersed droplets [17].

The organic solution of the essential oil obtained was dried with anhydrous sodium sulfate (Na_2_SO_4_), then weighed, and stored at 4°C in a tightly closed brown glass bottle to protect it from air and light.

### 2.3. Determination of the Essential Oil Yield

According to the AFNOR standard, the essential oil yield (EOY) is defined as the ratio between the mass of the essential oil obtained after extraction (*M*′) and the mass of the plant material used (*M*). It is given by the following formula [18]:

(1) $$\begin{array}{c} \text{EOY}\left(\%\right)=\left(\frac{M^{\prime }}{M}\right)*100, \end{array}$$

where EOY is the essential oil yield (in percent), *M*′ is the mass of the essential oil obtained in grams, and *M* is the mass of the plant material used in grams.

### 2.4. Phytochemical Analysis of *R*. *chalepensis* Essential Oil

The chromatographic analysis was carried out using a gas chromatograph (Thermo Scientific ISQ type) equipped with a DB-capillary column (30 m × 0.25 mm, film thickness: 0.25 *μ*m). The carrier gas is nitrogen with a flow rate of 15 ml/min. The device is equipped with a PVT (Programmed Vaporization Temperature) injector of the Split-Splitless type. The volume injected was 1 *μ*l. The temperature programming ranges from 40°C to 200°C with a gradient of 4°C/min.

The identification of the constituents was based on the comparison of their mass spectra (CPG/SM) with spectra from the library of the National Institute of Standards and Technology (NIST 98) and from the bibliography [19] and on the basis of the calculation of the Kováts index. The recorded spectra were processed using Xcalibur® software (version 1.4) from the company Thermo Fisher (Ulis, France).

### 2.5. Insecticidal Activity

#### 2.5.1. Repellent Effect of *R*. *chalepensis* Essential Oil on Filter Paper

The repellent effect of the essential oil of *R*. *chalepensis* L. on adults of *Tribolium castaneum* Herbst was evaluated using the preferential area method on filter paper described by McDonald's [20], under the same conditions as those used for mass rearing (a temperature of 30°C and relative humidity of 70%). As experimental containers, Petri dishes (9 cm in diameter) were used to confine the adult *Tribolium castaneum*. Filter paper discs (9 cm diameter) were divided into two equal parts. The three tested volumes of essential oil (3, 4, and 5 *μ*l) corresponding, respectively, to three concentrations (0.023, 0.031, and 0.038 *μ*l/ml) were deposited separately on one-half of the disc using a micropipette, as uniformly as possible. The concentration was calculated by relating the volume of the essential oil to the volume of the Petri dish (a 10.5 *∗* 1.5 cm dish provides 129.89 ml of air). The other half of the filter paper (control) received distilled water with the same volumes used for the essential oil. After air drying, each disc containing one part treated with the essential oil and the other with distilled water was carefully fixed to the bottom of a Petri dish. Ten nonsexed adult insects (with no separation between male and female sex) were released in the center of the filter paper disc and the Petri dish was then covered with a lid.

Three repetitions were used for each concentration. Counting of insects present on each part of the disc was carried out every 5 minutes for one hour; the percentage of repulsion (PR) was calculated using the following formula:

(2) $$\begin{array}{c} \mathrm{PR}\;\left(\%\right)=\left(\frac{Nc-Nt}{Nc+Nt}\right)*100, \end{array}$$

where *Nt* represents the number of insects present on the part of filter paper treated with essential oil and *Nc* represents the number of those present on the part treated only with water.

The average percentage of repulsion for the essential oil was calculated and assigned according to the McDonald classification, to one of the different repellent classes varying from 0 to V: class 0 (PR < 0.1%), class I (PR = 0.1–20%), class II (PR = 20.1–40%), class III (PR = 40.1–60%), class IV (PR = 60.1–80%), and class V (PR = 80.1–100%) [20].

#### 2.5.2. Inhalation Test Created in a Petri Dish

The study of the insecticidal activity was evaluated by several preliminary tests in order to choose the concentrations to be used for the evaluation of the insecticidal potential of *Ruta chalepensis* essential oil. The volumes used were 20, 40, 60, and 80 *μ*l/Petri dish, corresponding, respectively, to the concentrations 0.15; 0.31; 0.46, and 0.62 *μ*l/mL of air. Filter papers with a diameter of 4 cm were impregnated separately by the different volumes tested. Subsequently, the paper discs were attached to the lids of Petri dishes. The dishes were then covered after the introduction of 20 nonsexed adult insects (without separation between male and female sex) [21] aged between 10 and 14 days [15] into each Petri dish. The dishes were placed under the same environmental conditions as those used for insect rearing. The control dishes were prepared in the same way without the addition of essential oil [21].

Mortality control was done by counting dead insects every 12 hours for two days. Three replicates were performed for each dose and their average would represent the percentage of mortality.

The percentage of mortality observed in control and treated insects was calculated using the formula of Abbott [22]:

(3) $$\begin{array}{c} Pc=\frac{\left(Po-Pt\right)}{\left(100-Pt\right)}*100, \end{array}$$

where *Pc* is the mortality corrected in %, *Pt* is the mortality observed in the control, and *Po* is the mortality observed in the test.

#### 2.5.3. Calculation of LD_50_ and LT_50_

The results of the *Tribolium castaneum* adults mortality obtained after several exposure times and under the influence of several concentrations of *Ruta chalepensis* L. essential oil were used to calculate the LD_50_ (lethal dose which causes the death of half a population) and the TL_50_ (lethal time after which there is 50% mortality).

Indeed, to estimate the LD_50_ and the LT_50_, the percentages of mortality were transformed in probits and the concentrations and time in decimal logarithm. These transformations have allowed us to establish equations for regression lines “probit-logarithm” of type:

(4) $$\begin{array}{c} y=ax+b, \end{array}$$

where *y* is the corrected mortality probits, *x* is the logarithm of the dose or time, and *a* is the slope.

From the equations of the regression lines, the LD_50_ and the LT_50_ were calculated by replacing *y* by the probit of 50%. The LD_50_ and TL50 could also be determined graphically by looking for the abscissa of the point corresponding to probit 5; thus, the LD_50_ or TL_50_ would be equal to anti-log10*x* *μ*l/ml with *x* = log10 of the time or of the dose corresponding to probit 5 on the graph [23]. The results were obtained using GraphPad software (prism5 version 5.03 December 10, 2009) and Log-probit analysis software (WinDL version 2.0) developed by CIRAD-CA/MABIS performed by CIRAD-CA/MABIS.

#### 2.5.4. Repeated Factor and Response Variables

Table 1 presents the MANOVA parameters of the repeated measures for the main effects and the associated interactions between and within the variables.

## 3. Results

### 3.1. Yield of *R*. *chalepensis* L. Essential Oil

The essential oil extracted from the aerial part of *Ruta chalepensis* L. from the region of Oulmes presents a pale yellow color and has a strong odor; the yield obtained was about 2.72%.

### 3.2. Chemical Composition of *Ruta chalepensis* L. Essential Oil

**Figure 2:**
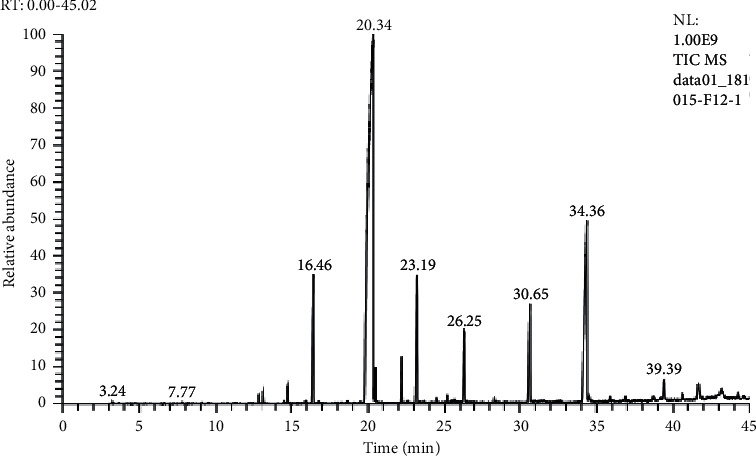
Chromatographic profile for GC-MS analysis of *Ruta chalepensis* L. essential oil.

The CG-SM analysis spectrum of the essential oil extracted from *Ruta chalepensis* growing in the Oulmes region is shown in Figure 2 and its detailed chemical composition is given in Table 2. Twenty constituents representing more than 95.93% of the overall composition of the essential oil have been identified, among which, six compounds representing approximately 92.15% of this oil have been identified as major constituents. These are 2-undecanone (64.35%), piperonyl piperazine (11.9%), 2-decanaone (5.12%), 2-dodecanone (4.52%), decipidone (3.9%), and 2-tridecanone (2.36%).

### 3.3. Repulsive Effect of *Ruta chalepensis* L. Essential Oil

**Figure 3:**
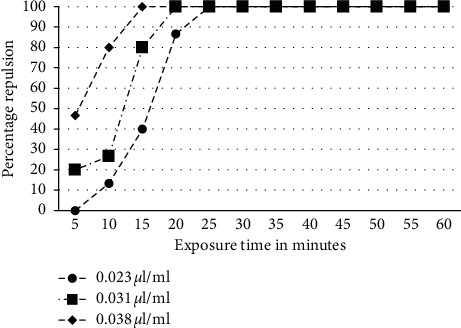
Repulsion percentage caused by the *Ruta chalepensis* essential oil relating to the exposure duration in mn.

**Figure 4:**
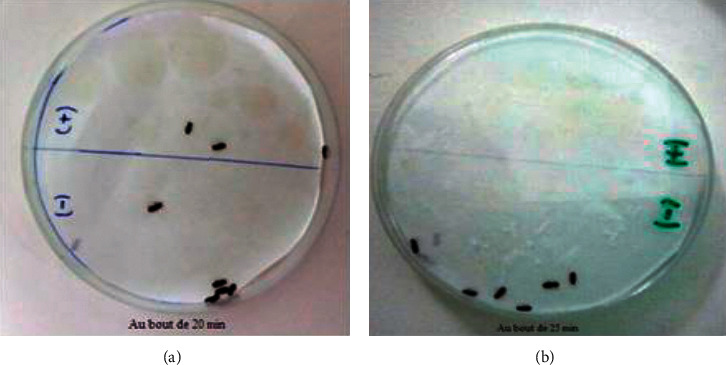
Results of the repellent test (case of the concentration of 0.023 *μ*l/ml).

The repulsion percentages obtained with some adults of *Tribolium castaneum* after one hour of their exposure to different doses of the *Ruta chalepensis* L. essential oil are summarized in Figure 3; it appears that the dose of 0.038 *μ*l/ml represents a significant repellency (100%) after 15 min; the 0.031 *μ*l/ml dose caused repulsion of all adults after 20 min of exposure while the dose of 0.023 *μ*l/ml had no repulsive effect until 10 min of exposure and 100% of repulsion was reached after 25 min Figure 4.

The analysis of variance (MANOVA at *P* < 0.05) revealed a significant effect of the *Ruta chalepensis* essential oil concentration and the duration of exposure tested on adults of *Tribolium castaneum*; the comparison of means was performed using the Tukey test at the 5% probability level, and it appears that the repellent activity of the essential oil was correlated with the concentrations and the duration of exposure (Table 3).

In columns, the comparison is made between the concentrations (lowercase letter). In rows, the comparison is made between the exposure times for each concentration (capital letter). The means followed by the same letter are not statistically different at *P* < 0.05.

### 3.4. Inhalation Test of *Ruta chalepensis* L. Essential Oil

**Figure 5:**
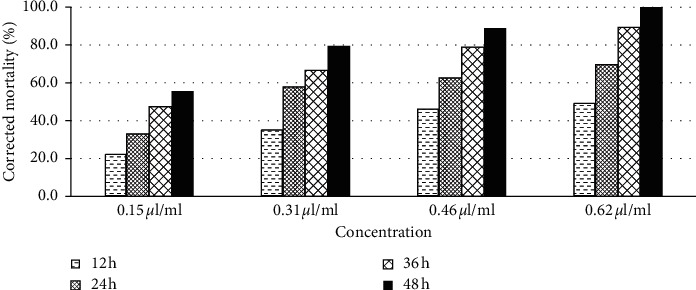
Percentage of the corrected mortality of *Tribolium castaneum* treated with the *Ruta chalepensis* essential oil.

Figure 5 illustrates the evolution of the corrected mortality rates of adults of *Tribolium castaneum* as a function of time and of the concentration of the *Ruta chalepensis* L. essential oil. It appears that the plant studied has a significant insecticide power as even at low concentrations of 0.15 *μ*l/ml, the percentage of mortality reached 55.56% after 48 hours of incubation. Furthermore, this period decreased when the dose used was increased; thus, for a dose of 0.31 *μ*l/ml, the mortality reached 57.90% after 24 hours.

### 3.5. Calculation of DL_50_ and TL_50_

**Figure 6:**
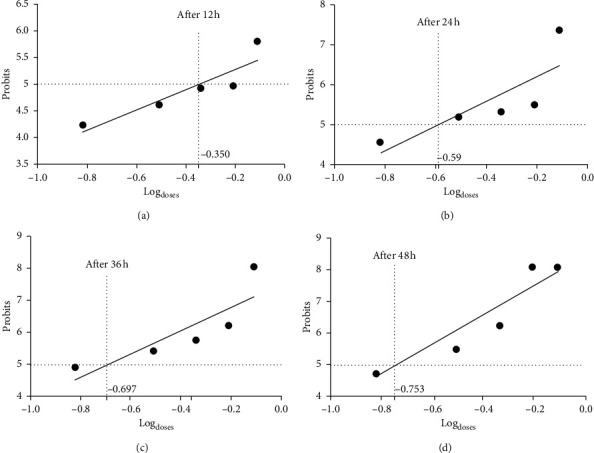
Efficacy of the 4 incubation periods on adults of *Tribolium castaneum* according to the concentration of the essential oil of *Ruta chalepensis* L. (a) Action of the oil after 12 hours. (b) Action of the oil after 24 hours. (c) Action of the oil after 36 hours. (d) Action of the oil after 48 hours.

The results concerning the LD_50_ values are represented in Figures 6(a)–6(d) and in Table 4. It should be noted that the concentrations of the essential oil of *Ruta chalepensis* which cause the mortality of 50% of the adults of *Tribolium castaneum* are, respectively, 0.447; 0.257; 0.20, and 0.176 *μ*l/ml for respective incubation times of 12, 24, 36, and 48 h.

**Figure 7:**
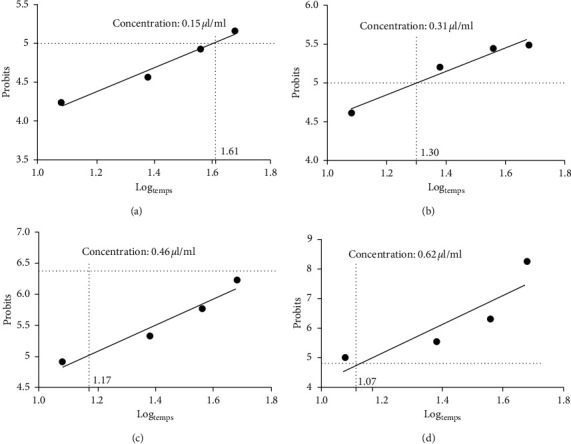
Efficacy of the 4 concentrations of the essential oil of *Ruta chalepensis* L. over time against adults of *Tribolium castaneum*. (a) Action of the oil at a concentration of 0.15 *μ*l/ml over time. (b) Action of the oil at a concentration of 0.31 *μ*l/ml over time. (c) Action of the oil at a concentration of 0.46 *μ*l/ml over time. (d) Action of the oil at a concentration of 0.62 *μ*l/ml over time.

The survival times of 50% of adults exposed to the different concentrations of essential oil range from 11.75 to 40.73 hours depending on the concentration (Figure 7 and Table 5).

## 4. Discussion

The essential oil yield of *R. chalepensis* from Oulmes (2.72%) was higher than that obtained by the same species collected from other regions, either in Morocco (1%) [24] or in central Tunisia (0.87%) [25] or that growing in the wild in Algeria (0.9%) [26]. The main compounds of the essential oil studied were 2-undecanone (64.35%), piperonyl piperazine (11.9%), 2-decanaone (5.12%), 2-dodecanone (4.52%), decipidone (3.9%), and 2-tridecanone (2.36%). The comparison of the results obtained with those of other studies on the same species but with different origins shows a difference in composition, more quantitatively than qualitatively. Indeed, for *Ruta chalepensis* L. growing in the wild in Tunisia, three main constituents have been reported: 2-undecanone (25.94%), 5-dodecanone acetate (9.35%), and 2-decanone (2.42%) [25]. Similarly, the work undertaken by [27] has shown that the essential oil of *Ruta chalepensis* collected from the north of Tunisia contains 2-undecanone (77.18%), 2-decanone (8.96%), and 2-dodecanone (2.37%) as major constituents. Furthermore, the major constituents of the Palestinian *Ruta chalepensis* essential oil were linalyl acetate (34.21%), *β*-linalool (31.78%), and 2-nonanone (8.15%) [28]. These differences observed in the chemical composition of *Ruta chalepensis* L. essential oil from different regions can be explained by several factors, particularly edaphic and climatic ones. The chemical profile of the essential oil is also influenced by the harvest period and stage and depends on the extraction and analysis techniques used [29].

Biological tests carried out on the adults of *Tribolium castaneum* have led to the conclusion that the essential oil of *Ruta chalepensis* L. collected from the Oulmes region has a very high repellent power belonging to the class V; also, when the dose used increases, the repellency rate increases and the duration of exposure decreases. Therefore, the repellent effect of the essential oil of *Ruta chalepensis* against the adults of *Tribolium castaneum* is dose- and time-dependent.

Furthermore, The repellent effect of the essential oil of *Ruta chalepensis* L. against *Tribolium castaneum* is much more important than that of certain oils studied in other research works, such as the essential oils of *Eucalyptus citriodora* and *Cymbopogon citratus*, which, with a concentration of 0.1 ml/l and an exposure time of 2 hours, gave PR = 76% and PR = 84%, respectively [30] whereas, with the essential oil of *Ruta chalepensis*, the PR is 86.76% with only a concentration of 0.023 ml/l and after 20 min.

It would seem that the insect repellency of the essential oil of *Ruta chalepensis* is due to its major compound, 2-undecanone (64.35%). Indeed, in 2007, in the United States of America, the Environmental Protection Agency approved the repellant BioUD®, in which the active compound is 2-undecanone (7.75%) isolated from wild tomatoes (*Lycopersicon hirsutum* Dunal f. *glabratum* C. H. Müll). BioUD® was more effective against mosquitos (*Aedes aegypti* L. and *Aedes albopictus* Skuse) and the American dog tick (*Dermacentor variabilis* Say.) than the commercial repellant DEET (N, N diethyl-m-toluamide), mostly used against arthropods [31], thus proving the strong repellent effect of 2-undecanone.

Moreover, *Ruta chalepensis* L. is characterized by its broad spectrum of action; in fact, other studies have shown a repellent effect of the essential oil of *Ruta chalepensis* L. against other types of insects belonging to the same genus of *Tribolium* such as *Tribolium confusum* [25] or other species such as *Rhyzopertha dominica* belonging to another genus [32].

Inhalation tests conducted have revealed the significant insecticidal power of essential oils of *Ruta chalepensis* on the adults of *Tribolium castaneum*; this mode of toxicity has been reported for several insect pests of stored food [33, 34] and is due to the volatile chemical compounds of the essential oils [35]. Thus, this insecticidal effect of the essential oils tested can be observed even at low concentrations. During the experiment, it was observed that incubation periods decrease when the doses used increase, which leads to the conclusion that the insecticidal effect of the essential oil of *Ruta chalepensis* against adults of *Tribolium castaneum* is closely dependent on the concentration used and is, therefore, dose-dependent or dose/effect.

On the one hand, this insecticidal power is in line with the results obtained when using *Ruta chalepensis* powder on the same insect [7]; on the other hand, negative results were obtained with the use of crude extracts of *Ruta chalepensis* obtained by maceration, decoction, and infusion [7]. Thus, the observed fumigant activity demonstrates that essential oils rich in volatile compounds are a source of biologically active vapors that can potentially prove to be effective insecticides as opposed to crude extracts. The toxicity of *Ruta chalepensis* essential oil to *Tribolium castaneum* can be attributed to its high content of 2-undecanone, an aliphatic ketone with previously demonstrated insecticidal properties [36].

The tests carried out revealed that the concentrations of essential oil of *Ruta chalepensis* which cause the mortality of 50% of the adults of *Tribolium castaneum* are, respectively,0.447, 0.257, 0.20, and 0.176 *μ*l/ml for incubation periods of 12, 24, 36, and 48 hours, respectively. It is clearly observable that the longer the exposure time, the lower the dose required to kill half of the target population; therefore, the exposure time of insects to the essential oil is negatively correlated with the LD_50_ (*R*^2^ = −1).

On the other hand, the essential oil of another plant *Thymus leptobotrys* applied to the same insect showed higher toxicity, with an LD_50_ of 0.08 *μ*l/ml [36].

Concerning the survival times of 50% of adults exposed to different concentrations of essential oil, it was found that they vary from 11.75 to 40.73 hours depending on the concentration. The TL_50s_ are negatively correlated with the concentrations of the essential oil tested (*R*^2^ = −1). In another study, TL_50_ values ranging from 32.80 to 69.37 hours were observed for the essential oil of *Thymus leptobotrys* tested against *Tribolium castaneum* [36]. Such variability of the TL_50_ and LD_50_ can only be explained by the variation of the chemical profiles of the tested essential oils [37]. Taking into account the origin, it appears that after 24 hours, mortality reaches 60% for a dose of 0.2 *μ*l/ml with an LD_50_ of about 0.176 *μ*l/ml for essential oil of *Ruta chalepensis* from Mednine (Tunisia) [38]; keeping the same exposure time, almost this mortality rate is reached with a dose of 0.31 *μ*l/ml and an LD_50_ = 0.257 *μ*l/ml for the essential oil of *Ruta chalepensis* from the study region Oulmes (Morocco). Thus, the origin influences the fumigant toxicity effect of *Ruta chalepensis* essential oil. This variation in the insecticidal effect linked to the plant's origin can be explained by the variation in the chemical composition of the plant and/or the content of active compounds. The two major compounds of *Ruta chalepensis* essential oil from Mednine (Tunisia) are 2-undecanone (48.28%) and 2-nonanone (27.15%) [38], while for the one from Oulmes (Morocco) they are 2-undecanone (64.35%) and piperonyl piperazine (11.9%).

Thus, given the molecular diversity of the essential oil of *Ruta chalepensis* L. (Table 2), it seems more likely that its insecticidal activity results from the association of several mechanisms which are exerted jointly on different target parts of *Tribolium castaneum*. In addition, the rapid action of essential oils on harmful insects is due to a neurotoxic effect; indeed, essential oils can penetrate through the cuticle and come into contact with the nerve endings of the trachea of the insect and then cause neurotoxic activity and rapid death [39]. Symptoms induced by the volatile forms of these natural compounds applied by inhalation are generally similar to those seen in topical or contact bioassays, suggesting a common mode of action despite different routes of exposure [40].

The neurotoxic modes of action on insects are mainly linked to the enzymatic activity of acetylcholinesterase [41]. For their part, studies carried out on the effects of the essential oil of *Ruta chalepensis* L. on the species of the genus *Tribolium* such as *Tribolium confusum* have shown that it induces glutathione S-transferases and reduces the activity of the acetylcholinesterase [25]. The glutathione S-transferases are involved in insecticide resistance [42] and play a role in the metabolism of organophosphorus and organochlorine compounds [43]. The reduction of the acetylcholinesterase by many neurotoxicants causes the accumulation of acetylcholine in the synaptic space which maintains a permanent transmission of nerve impulses; the action of the essential oil of *Ruta chalepensis* L. is manifested by convulsions, lack of coordination of movements and tremors [25].

In addition, from a physicochemical point of view, essential oils leave no residue or waste in the treated products and are easily biodegradable [44]. They are, therefore, potentially eligible for the development of new classes of insecticides combining efficiency and protection of the environment [45, 46]. Regarding application in storage warehouses, one of the most appreciated properties of essential oils is their fumigant activity against insects, since their involvement can be successful without having to apply the compound directly to the insects [9].

## 5. Conclusions

This study was carried out in order to determine the chemical composition of *Ruta chalepensis* essential oil from the region of Oulmes (Central plateau of Morocco) and to evaluate these effects in two bioassays: repellent and fumigant activities against a major stored grain pest *Tribolium castaneum*. Chromatographic analyses revealed the richness of this oil in volatile secondary metabolites including 2-undecanone (64.35%) which seems to be responsible for its insect repellent and insecticidal properties. Indeed, the essential oil of *Ruta chalepensis* from the region of Oulmes has a very high repellent power which reaches 100% after only 15 min with a dose not exceeding 0.038 *μ*l/ml. Inhalation tests have also revealed the important insecticidal effect of this oil even at low doses. These tests revealed the efficiency of the essential oil of *Ruta chalepensis* L. against the adults of *Tribolium castaneum*, which proves that this natural substance could be a healthy alternative to synthetic products to fight this pest, especially as its fumigant activity can be invested without a direct application on stored foodstuffs. Also, a thorough study of the insecticidal activity of this essential oil against *Tribolium castaneum* at different stages of development would make it possible to guarantee the proper storage of cereal products.

## Figures and Tables

**Table 1:** Repeated factor and response variables.

	*df*	*F* value	*P* value
Tests of within-subjects effects			
Temps	1.949	114.821	0.000
Temps *∗* concentration	3.898	8.514	0.002

Tests of between-subjects effects			
Intercept	1	2521.869	0.000
Concentration	2	66.902	0.000

**Table 2:** Chemical composition of the essential oil of *Ruta chalepensis* L.

No.	TR (min)	Constituent	*IK*	%
1	12.78	2-Nonanone	1028	0.2
2	13.17	Nonanal	1082	0.28
3	14.73	Geijerene	1087	0.43
4	15.95	1-Nonanol	1099	0.09
5	16.46	2-Decanaone	1162	5.12
6	16.80	Decanal	1285	0.05
7	20.34	2-Undecanone	1293	64.35
8	23.19	2-Dodecanone	1361	4.52
9	24.46	2-Hexanone 6-phenyl	1372	0.14
10	26.25	2-Tridecanone	1485	2.36
11	28.33	2-Tetradecanone	1491	0.13
12	30.65	Decipidone	1522	3.9
13	34.36	Piperonyl piperazine	1567	11.9
14	35.87	2-Pentadecanone	1601	0.13
15	36.86	Cyclononasiloxane octadecamethyl	1609	0.17
16	38.71	n-Hexadecanoic acid	1634	0.14
17	40.05	Octadecane	1657	0.6
18	40.66	Cyclodecasiloxane eicosamethyl	1673	0.5
19	41.66	Levoglucosan	1698	0.76
20	44.60	Tetracosane	1751	0.16
Total				95.93

TR: retention time (min), IK: Kováts index.

**Table 3:** Percentage of repulsion (means ± standard deviation) of the essential oil of *Ruta chalepensis* L. against adults of *Tribolium castaneum* after various exposure periods.

Concentration (*μ*l/ml)	5 min	10 min	15 min	20 min	25 min	d*f*	*F*	*P*
0.023	00 ± 00 aA	13.33 ± 11.55 aA	40 ± 20 aB	86.67 ± 11.55 aC	100 ± 00 aC	4	43.678	0.000
0.031	20 ± 00 bA	26.67 ± 11.55 aA	80 ± 00 bB	100 ± 00 aC	100 ± 00 aC	4	173.198	0.000
0.038	46.67 ± 11.55 cA	80 ± 20 bB	100 ± 00 bB	100 ± 00 aB	100 ± 00 aB	4	15.238	0.000
d*f*	2	2	2	2	2			
*F*	36.957	16.788	21.00	3.988				
*P*	0.000	0.003	0.002	0.07				

**Table 4:** Equation of regression lines, regression coefficients, and lethal dose 50 (LD_50_) values evaluated for the 4 incubation durations tested.

Time (h)	Equation of regression	*R* ^2^	Log_dose_	DL50 (*μ*l/ml)
12	*Y* = 1.8787*x* + 5.6561	0.8126	−0.349	0.447
24	*Y* = 3.0405*x* + 6.7947	0.6485	−0.590	0.257
36	*Y* = 3.5151*x* + 7.4497	0.7067	−0.697	0.201
48	*Y* = 4.5358*x* + 8.4142	0.8156	−0.753	0.176

**Table 5:** Equation of regression lines, regression coefficients, and lethal time 50 (LT_50_) values evaluated for the 4 concentrations of *Ruta chalepensis* essential oil.

Concentration (*μ*l/ml)	Equation of regression	*R* ^2^	Log time	TL_50_ (h)
0.15	*Y* = 1.5311*x* + 2.5348	0.9770	1.610	40.73
0.31	*Y* = 1.5026*x* + 3.0425	0.9559	1.303	19.95
0.46	*Y* = 2.1074*x* + 2.5261	0.9503	1.174	14.79
0.62	*Y* = 2.8117*x* + 1.9839	0.7830	1.073	11.75

## Data Availability

The data sets used and/or analyzed during the current study are available from the corresponding author upon reasonable request.
